# Antimicrobial Activity of Zinc Oxide Nanoparticles Synthesized Using Ocimum Tenuiflorum and Ocimum Gratissimum Herbal Formulation Against Oral Pathogens

**DOI:** 10.7759/cureus.53562

**Published:** 2024-02-04

**Authors:** Remmiya Mary Varghese, Aravind Kumar S, Rajeshkumar Shanmugam

**Affiliations:** 1 Orthodontics and Dentofacial Orthopaedics, Saveetha Dental College and Hospitals, Saveetha Institute of Medical and Technical Sciences, Saveetha University, Chennai, IND; 2 Orthodontics and Dentofacial Orthopedics, Saveetha Dental College and Hospitals, Saveetha Institute of Medical and Technical Sciences, Saveetha University, Chennai, IND; 3 Nanobiomedicine Lab, Centre for Global Health Research, Saveetha Medical College Hospital, Saveetha Institute of Medical and Technical Sciences, Chennai, IND; 4 Pharmacology, Saveetha Dental College and Hospitals, Saveetha Institute of Medical and Technical Sciences, Saveetha University, Chennai, IND

**Keywords:** nanotechnology, antimicrobial agent, ecofriendly, green synthesis, zinc oxide nanoparticle

## Abstract

Background

This study deals with the antimicrobial efficacy of zinc oxide nanoparticles (ZnONPs) synthesized through green methods employing extracts from *Ocimum tenuiflorum and Ocimum gratissimum* and assessed for their antimicrobial properties against a range of oral pathogens.

Methods

Zinc oxide nanoparticles (ZnONPs) were synthesized using extracts from *Ocimum tenuiflorum* and *Ocimum gratissimum* through a green synthesis approach. Antimicrobial activity was determined using the agar-well diffusion assay to evaluate the consistency of inhibition zones against oral pathogens. Variations in sensitivity were assessed through the time-kill curve assay, quantifying the response of oral pathogens to zinc oxide nanoparticles (ZnONPs) exposure over time.

Results

The agar-well diffusion assay revealed uniform 9-mm zones of inhibition against all oral pathogens, signifying consistent antimicrobial activity of zinc oxide nanoparticles (ZnONPs). In the time-kill curve assay, Candida albicans exhibited the highest sensitivity, followed by *Streptococcus mutans and Staphylococcus aureus. Enterococcus faecalis and Lactobacillus species* displayed lower sensitivity, suggesting potential selectivity.

Discussion

The observed variation in sensitivity implies the potential selectivity of zinc oxide nanoparticles (ZnONPs) against specific oral pathogens, which may have significant implications for oral health applications. These findings underscore the versatility of green-synthesized zinc oxide nanoparticles (ZnONPs) as promising antimicrobial agents, particularly for oral health applications.

Conclusion

This study provides promising results for the antimicrobial potential of zinc oxide nanoparticles (ZnONPs) synthesized using *Ocimum tenuiflorum and Ocimum gratissimum*. The consistent antimicrobial activity and variations in sensitivity among oral pathogens highlight their promising utility in oral health care.

## Introduction

In recent years, there has been a growing interest in leveraging natural products and nanotechnology for the creation of innovative antimicrobial agents [1]. Among these natural products, medicinal plants have garnered significant attention due to their long-standing use in traditional medicine and their potential therapeutic properties. Two such plants, *Ocimum tenuiflorum*, commonly known as Holy Basil or Tulsi, and *Ocimum gratissimum*, also known as African Basil or Clove Basil, have been recognized for their medicinal value [2,3].

Tulsi, a revered herb in Ayurveda, possesses a rich history of medicinal applications, including the treatment of respiratory issues, skin ailments, and digestive disorders [4]. It owes its therapeutic attributes to bioactive compounds like eugenol, rosmarinic acid, and ursolic acid, which confer antimicrobial, anti-inflammatory, and antioxidant properties [5]. African basil, native to Africa, is celebrated for its robust fragrance and taste, courtesy of essential oils like eugenol, thymol, and methyl chavicol. This plant has found extensive use in traditional African medicine, addressing conditions such as respiratory infections, skin maladies, and gastrointestinal disorders [6].

Zinc oxide nanoparticles (ZnONPs) have gained prominence owing to their unique characteristics and diverse applications, including medicine. ZnONPs have been thoroughly investigated for their antimicrobial prowess against an array of pathogens encompassing bacteria, fungi, and viruses [7]. The antimicrobial mechanism of ZnONPs is rooted in the release of zinc ions, disrupting cell membranes and impeding microorganism growth [8]. Moreover, ZnONPs exhibit anti-inflammatory and antioxidant attributes, positioning them as promising contenders for antimicrobial agent development [9].

Oral pathogens, comprising bacteria and fungi, exert a pivotal role in the onset of oral afflictions such as dental caries, periodontitis, and oral candidiasis [10]. The emergence of drug-resistant strains among these pathogens has posed a formidable challenge in oral healthcare. Thus, there exists an urgent need to explore alternative antimicrobial agents capable of effectively combating these oral pathogens [11,12].

The amalgamation of herbal formulations with nanoparticles presents a potential avenue to amplify the antimicrobial potential of natural substances [13]. The synergy between bioactive compounds inherent to medicinal plants and the distinctive properties of nanoparticles may yield more potent antimicrobial agents. In the case of *Ocimum tenuiflorum* and *Ocimum gratissimum*, the integration of zinc oxide nanoparticles into their herbal formulations holds the promise of enhancing their antimicrobial efficacy against oral pathogens [14,15].

This study's objective is to assess the antimicrobial activity of *Ocimum tenuiflorum* and *Ocimum gratissimum* herbal formulation-mediated zinc oxide nanoparticles against oral pathogens. The investigation involves determining the zone of inhibition via the agar-well diffusion technique and assessing sensitivity variations through the time-kill curve assay of the herbal formulation-mediated zinc oxide nanoparticles (ZnONPs). The findings anticipated from this study are poised to make substantial contributions to the development of alternative antimicrobial agents for the treatment and prevention of oral diseases. The amalgamation of natural products and nanotechnology presents a promising avenue to address the challenges posed by drug-resistant oral pathogens. By harnessing the therapeutic potential of *Ocimum tenuiflorum* and *Ocimum gratissimum* and coupling it with the antimicrobial attributes of zinc oxide nanoparticles, this research has the potential to yield valuable insights into the creation of efficacious interventions for oral healthcare.

## Materials and methods

Ethical approval for this study was obtained from the Ethical Committee of the Saveetha University, Saveetha Institute of Medical and Technical Sciences, Chennai (Ethical Number: SIMATS/Ph.D. Regn/A4/ 2O2O/0597-01). This research was conducted ethically, following the guidelines approved by the ethical committee.

Preparation of herbal formulations

1g of *Ocimum tenuiflorum* and 1g of *Ocimum gratissimum* were accurately added to 100mL of distilled water. The mixture was then subjected to heating using a heating mantle at a temperature of 60°C for a duration of 15-20 minutes. Subsequently, the boiled mixture underwent a gradual filtration process utilizing filter paper. The resulting filtrate, containing the extract, was then stored for subsequent nanoparticle synthesis.

Green synthesis of zinc oxide nanoparticles (ZnONPs)

The synthesis of zinc oxide nanoparticles (ZnONPs) using a green approach involving African basil and black tulsi (*Ocimum* *tenuiflorum* and *Ocimum sanctum*) extracts in the presence of zinc nitrate solution (30 mM in 50 mL distilled water) was undertaken in this study. This environmentally friendly method harnesses the bioactive compounds present in these herbal extracts for the reduction and stabilization of zinc oxide nanoparticles (ZnONPs). The procedure began with the preparation of a zinc nitrate solution, providing a controlled source of zinc ions. Subsequently, 50 mL of African basil and black tulsi extract, known for their rich phytochemical content, was combined with the zinc nitrate solution. The resulting mixture was subjected to a centrifugation process at 8000 rpm for 10 minutes. The centrifugation step played a pivotal role in the zinc oxide nanoparticles (ZnONPs) synthesis process. It facilitated the separation of synthesized nanoparticles from any unreacted precursors or extract residues. The pellet collected after centrifugation represents the desired zinc oxide nanoparticles (ZnONPs), which were then subjected to further characterization and evaluation.

Antimicrobial activity

The agar-well diffusion method, a commonly used technique, was employed to evaluate the antimicrobial activity of green-mediated zinc oxide nanoparticles against a panel of oral pathogens comprising *Streptococcus mutans, Enterococcus faecalis, Staphylococcus aureus, Lactobacillus, *and *Candida albicans*. The procedure involved preparing Mueller Hinton agar (MHA) plates as a growth medium, culturing the target bacteria in Mueller Hinton Broth (MHB) after 24 hours of incubation, and standardizing bacterial loads using McFarland standards. The bacterial cultures were uniformly swabbed onto MHA plates, followed by well creation and loading with green-mediated zinc oxide nanoparticles at various concentrations (25, 50, and 100 micrograms). After incubation at 37°C for 24 hours, the zones of inhibition were measured to gauge antimicrobial effectiveness. Comparative analysis with standard antibiotics provided insights into the nanoparticles' relative efficacy. Despite some limitations, including diffusion variability and a potentially incomplete representation of antimicrobial potential, the agar-well diffusion method remains a valuable initial screening tool for assessing antimicrobial properties.

Time-kill curve assay

The Time-Kill Kinetics Assay is a laboratory technique utilized to assess the bactericidal activity of antimicrobial agents against a range of oral pathogens, comprising *Streptococcus mutans, Enterococcus faecalis, Staphylococcus aureus, Lactobacillus*, and *Candida albicans*. In this assay, each bacterial strain is cultured individually, and they are exposed to varying concentrations of green-mediated zinc oxide nanoparticles (25, 50, and 100 micrograms) at specified time intervals (1 to 5 hours). Control groups without ZnONPs and treated with the plant extracts (*Ocimum tenuiflorum* and *Ocimum gratissimum* herbal formulations) are also included for comparison. Optical density measurements, reflecting the viable bacterial population, are taken using an ELISA plate reader, which was measured at 600 nm.

## Results

Antimicrobial activity

**Figure 1:**
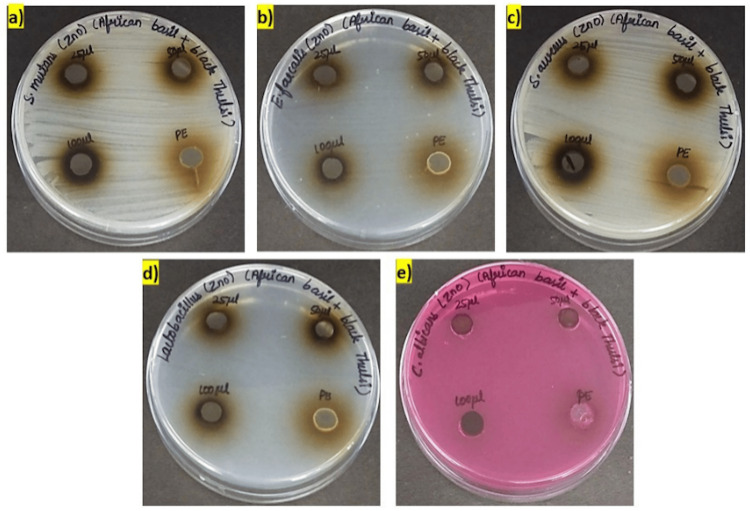
Antimicrobial activity of green synthesized zinc oxide nanoparticles against oral pathogens a) S. mutans b) E. faecalis c) S. aureus d) Lactobacillus e) C. albicans The zinc oxide nanoparticles (ZnONPs) were tested at three different concentrations 25 µg/mL, 50 µg/mL, and 100 µg/mL. PE : Plant extract

**Figure 2:**
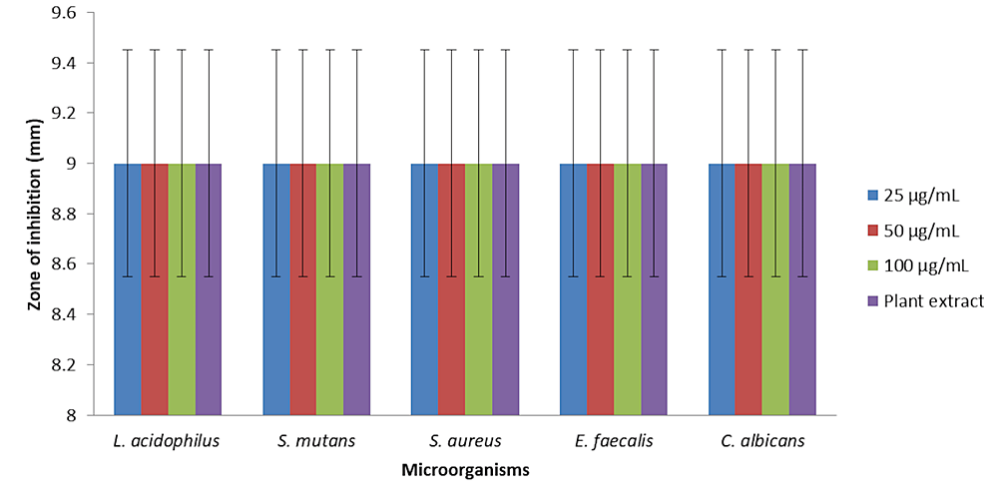
Agar-well diffusion technique to assess the antimicrobial activity of Ocimum tenuiflorum and Ocimum gratissimum-mediated zinc oxide nanoparticles.

In Figures 1, 2, the antimicrobial activity of green-synthesized zinc oxide nanoparticles (ZnONPs) was assessed against a panel of oral pathogens, comprising *Streptococcus mutans (S. mutans*), *Enterococcus faecalis (E. faecalis), Staphylococcus aureus (S. aureus), Lactobacillus acidophilus (L. acidophilus*), and* Candida albicans (C. albicans)*. The zinc oxide nanoparticles were tested at three different concentrations (25 µg/mL, 50 µg/mL, and 100 µg/mL), and the results were compared with those obtained using a plant extract as a control.

At all tested concentrations of zinc oxide nanoparticles (25 µg/mL, 50 µg/mL, and 100 µg/mL), there was no significant difference in the zone of inhibition observed for the tested microorganisms when compared to the plant extract control. All microorganisms, *S. mutans, E. faecalis, S. aureus, L. acidophilus*, and *C. albicans*, exhibited a zone of inhibition of 9 mm, regardless of the ZnONP concentration.

These findings suggest that the green-synthesized zinc oxide nanoparticles did not exhibit significant antimicrobial activity against the tested oral pathogens under the conditions tested. Further investigations may be needed to optimize the synthesis process or assess the nanoparticle efficacy under different experimental conditions to potentially enhance their antimicrobial properties.

Time-kill curve assay

**Figure 3:**
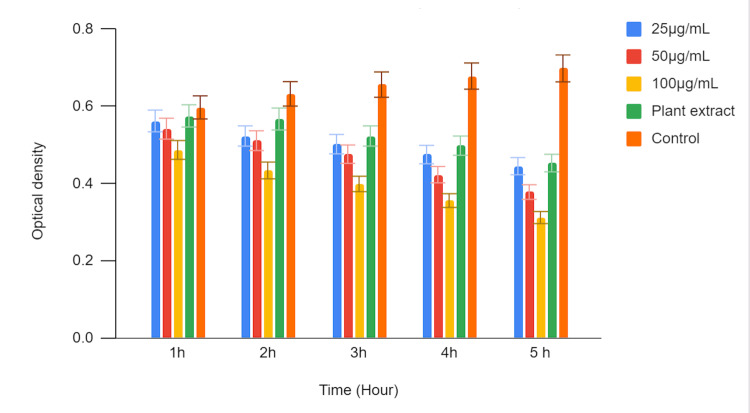
Time-kill curve assay of green-mediated zinc oxide nanoparticles against C. albicans

In Figure 3, the time-kill curve assay was conducted to evaluate the antifungal activity of green-mediated zinc oxide nanoparticles (ZnONPs) against *Candida albicans (C. albicans)*. Three different concentrations of zinc oxide nanoparticles (ZnONPs) (25 µg/mL, 50 µg/mL, and 100 µg/mL) were tested, and the results were compared with those obtained using a plant extract and a control. The optical density (OD) values were measured at various time points (1 hour, 2 hours, 3 hours, 4 hours, and 5 hours) to assess the growth inhibition of *C. albicans*.

At all tested time points, the optical density values (OD) were recorded for each concentration of zinc oxide nanoparticles, the plant extract, and the control. The lower OD values indicate a higher level of growth inhibition, reflecting the antifungal activity of the tested substances against *C. albicans.*

The optical density values (OD) gradually decreased over time, indicating a time-dependent inhibition of *C. albicans* growth. At 1 hour, the OD was 0.562, which decreased to 0.445 at 5 hours. Similar to the 25 µg/mL concentration, the OD values decreased over time, demonstrating a time-dependent inhibitory effect. The highest concentration of zinc oxide nanoparticles exhibited the most significant antifungal activity. The plant extract also showed antifungal activity, with an OD of 0.575 at 1 hour. The control group, representing untreated *C. albicans*, showed an increase in OD over time, indicating unrestricted growth. The OD values ranged from 0.597 at 1 hour to 0.698 at 5 hours.

The time-kill curve assay results demonstrate that green-mediated zinc oxide nanoparticles (ZnONPs) have antifungal activity against *Candida albicans* in a time- and concentration-dependent manner. Higher zinc oxide nanoparticle concentrations (50 µg/mL and 100 µg/mL) displayed stronger inhibitory effects on *C. albicans* growth compared to the lower concentration (25 µg/mL) and the plant extract. The control group, in contrast, exhibited continuous growth of *C. albicans *over the observed time period. These findings suggest the potential of green-mediated zinc oxide nanoparticles (ZnONPs) as an effective antifungal agent against* C. albicans* infections, demanding further investigation for potential therapeutic applications.

**Figure 4:**
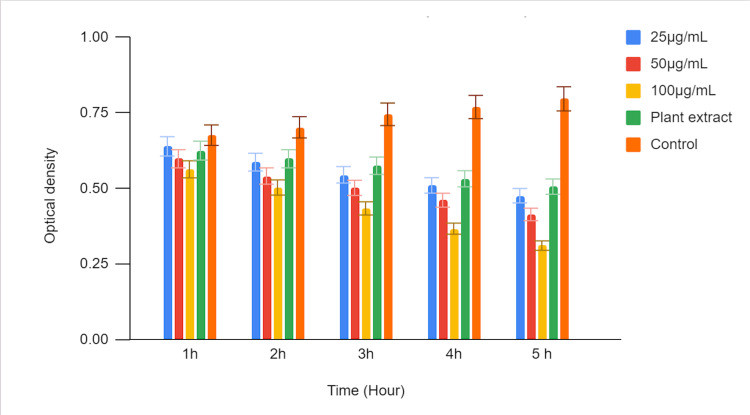
Time-kill curve assay of green-mediated zinc oxide nanoparticles against E. feacalis

In Figure 4, a time-kill curve assay was conducted to investigate the antimicrobial activity of green-mediated zinc oxide nanoparticles against *Enterococcus faecalis (E. faecalis)*. Three different concentrations of zinc oxide nanoparticles (25 µg/mL, 50 µg/mL, and 100 µg/mL) were evaluated, and their effects were compared to those of a plant extract and a control. Optical density (OD) values were measured at multiple time points (1 hour, 2 hours, 3 hours, 4 hours, and 5 hours) to assess the growth inhibition of *E. faecalis. *The recorded OD values at each time point represent the growth status of *E. faecalis.* Lower OD values indicate greater inhibition of bacterial growth, reflecting the antimicrobial activity of the tested substances against *E. faecalis*.

The highest concentration of zinc oxide nanoparticles exhibited the most substantial antimicrobial activity. The OD value was 0.563 at 1 hour and significantly decreased to 0.311 at 5 hours. The plant extract also displayed antimicrobial activity, with an OD of 0.625 at 1 hour, decreasing to 0.506 at 5 hours. The control group, representing untreated *E. faecalis,* showed an increase in OD over time, indicating unrestricted bacterial growth. 

The results of the time-kill curve assay demonstrate that green-mediated zinc oxide nanoparticles possess significant antimicrobial activity against *Enterococcus faecalis (E. faecalis)* in a time- and concentration-dependent manner. Higher zinc oxide nanoparticles (ZnONP) concentrations (50 µg/mL and 100 µg/mL) exhibited stronger bactericidal effects compared to the lower concentration (25 µg/mL) and the plant extract control. In contrast, the untreated control group showed continuous growth of *E. faecalis *over the observation period.

**Figure 5:**
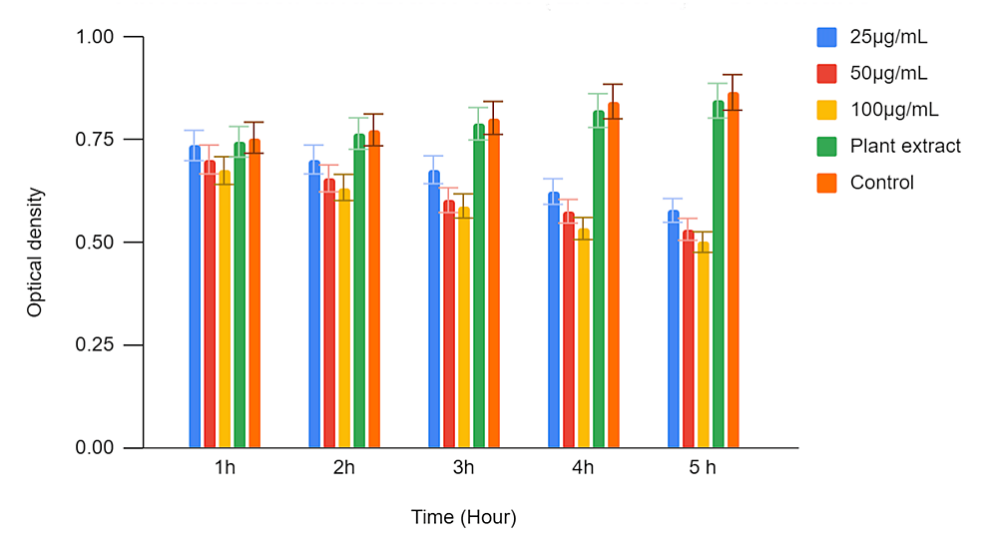
Time-kill curve assay of zinc oxide nanoparticles against S. mutans

In Figure 5, a time-kill curve assay was conducted to assess the antimicrobial activity of zinc oxide nanoparticles against *Streptococcus mutans (S. mutans)*. Three different concentrations of zinc oxide nanoparticles (25 µg/mL, 50 µg/mL, and 100 µg/mL) were tested, and their effects were compared to those of a plant extract and a control. Optical density (OD) values were measured at various time points (1 hour, 2 hours, 3 hours, 4 hours, and 5 hours) to evaluate the inhibition of *S. mutans* growth.

The recorded OD values at each time point represent the growth status of *S. mutans*. Lower OD values indicate stronger inhibition of bacterial growth, reflecting the antimicrobial activity of the tested substances against *S. mutans. *Over time, the OD values gradually decreased, indicating a time-dependent inhibition of *S. mutans* growth. Similar to the 25 µg/mL concentration, the OD values decreased over time, demonstrating time-dependent bacteriostatic effects. 

The highest concentration of zinc oxide nanoparticles (ZnONPs) exhibited significant antimicrobial activity. The plant extract also displayed antimicrobial activity, with an OD of 0.745 at 1 hour, increasing slightly before declining to 0.845 at 5 hours. The control group, representing untreated *S. mutans*, showed an increase in OD over time, indicating unrestricted bacterial growth. 

The time-kill curve assay results demonstrate that ZnONPs have significant antimicrobial activity against *Streptococcus mutans (S. mutans)* in a time- and concentration-dependent manner. Higher zinc oxide nanoparticle concentrations (50 µg/mL and 100 µg/mL) exhibited stronger inhibitory effects on *S. mutans* growth compared to the lower concentration (25 µg/mL) and the plant extract control. In contrast, the untreated control group showed continuous growth of *S. mutans* over the observation period. These findings highlight the potential of ZnONPs as effective antimicrobial agents against *S. mutans *infections and suggest their potential utility in oral health applications.

**Figure 6:**
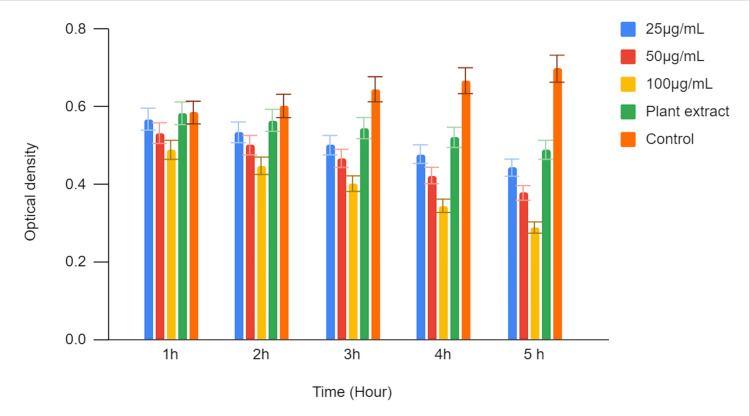
Time-kill curve assay of zinc oxide nanoparticles against S. aureus

In Figure 6, the time-kill curve assay of zinc oxide nanoparticles (ZnONPs) against *Staphylococcus aureus (S. aureus*), it was observed that all tested concentrations of zinc oxide nanoparticles (25 µg/mL, 50 µg/mL, and 100 µg/mL) exhibited time-dependent antimicrobial activity. The OD values progressively decreased over a five-hour period, indicating inhibition of *S. aureus* growth. The highest concentration of zinc oxide nanoparticles (100 µg/mL) demonstrated the most significant bactericidal effects. The plant extract also displayed antimicrobial activity, with a similar trend of decreasing OD values over time. In contrast, the untreated control group showed continued growth of *S. aureus.* These findings suggest that zinc oxide nanoparticles possess promising antimicrobial properties against *S. aureus*, with higher concentrations exhibiting stronger inhibition, which may have potential implications for therapeutic application.

**Figure 7:**
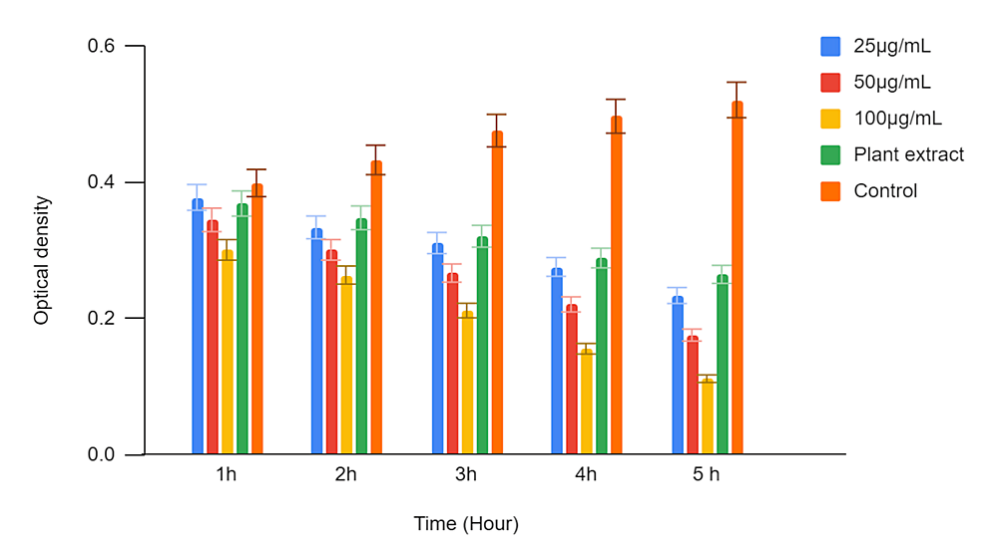
Time-kill curve assay of zinc oxide nanoparticles against Lactobacillus sp.

In Figure 7, the time-kill curve assay of zinc oxide nanoparticles against *Lactobacillus species (Lactobacillus sp.)*, the results revealed a notable concentration and time-dependent antimicrobial activity. All tested concentrations of ZnONPs (25 µg/mL, 50 µg/mL, and 100 µg/mL) exhibited a gradual reduction in optical density (OD) values over a 5-hour period, indicating a progressive inhibition of *Lactobacillus growth.* The highest concentration of zinc oxide nanoparticles (100 µg/mL) demonstrated the most substantial bactericidal effects, with an OD decrease from 0.501 at 1 hour to 0.323 at 5 hours. The plant extract also exhibited antimicrobial activity, with OD values decreasing over time in a manner similar to the zinc oxide nanoparticle treatments. In contrast, the untreated control group showed a consistent increase in OD values, indicating unimpeded growth of *Lactobacillus*. These findings underscore the potential of ZnONPs as effective antimicrobial agents against *Lactobacillus *species*,* with higher concentrations displaying more pronounced inhibition.

In summary, *Candida albicans* was the most sensitive organism to zinc oxide nanoparticles, followed by *Streptococcus mutans* and *Staphylococcus aureus*, which exhibited moderate sensitivity. *Enterococcus faecalis *and *Lactobacillus species* were relatively less sensitive to zinc oxide nanoparticles, requiring higher concentrations for significant inhibition. These variations in sensitivity highlight the importance of considering both concentration and the specific microorganism when using zinc oxide nanoparticles as antimicrobial agents.

## Discussion

The study aimed to assess the antimicrobial activity of zinc oxide nanoparticles (ZnONPs) synthesized through an herbal formulation involving *Ocimum tenuiflorum* (Holy Basil) and *Ocimum gratissimum* (African Basil) against a panel of oral pathogens. In addition to the time-kill curve assay, the antimicrobial activity was evaluated using the agar-well diffusion method, providing a comprehensive understanding of the zinc oxide nanoparticles (ZnONPs) efficacy. The results from the agar-well diffusion assay further corroborated the antimicrobial potential of green-synthesized zinc oxide nanoparticles. The assay demonstrated the ability of zinc oxide nanoparticles to create zones of inhibition around the wells, indicative of their antimicrobial effects [16]. Notably, the zones of inhibition observed for various oral pathogens were consistent with the concentration-dependent activity observed in the time-kill curve assay.

The comparative analysis revealed variations in the sensitivity of these oral pathogens to the zinc oxide nanoparticles. *C. albicans*, a fungal pathogen, exhibited the highest sensitivity to zinc oxide nanoparticles, with significant growth inhibition observed even at lower concentrations. This finding suggests the potential of zinc oxide nanoparticles in managing fungal oral infections, such as oral candidiasis [17].

*S. mutans* and* S. aureus,* which are common bacterial pathogens associated with dental caries and oral infections, displayed moderate sensitivity to zinc oxide nanoparticles. While zinc oxide nanoparticles effectively inhibited their growth in a time-dependent manner, higher ZnONP concentrations were required for pronounced effects. This implies that zinc oxide nanoparticles may have promise in controlling bacterial oral infections but may need optimization for maximal efficacy [18]. In contrast, *E. faecalis* and *Lactobacillus *species exhibited lower sensitivity to zinc oxide nanoparticles, necessitating higher concentrations for significant inhibition. *E. faecalis* is known for its association with endodontic infections, while *Lactobacillus *speciesinclude probiotics and commensal bacteria important for oral health. The lower sensitivity of these organisms suggests that zinc oxide nanoparticles may selectively target pathogenic bacteria while sparing beneficial ones, a crucial consideration in maintaining oral microbiome balance. The combined results of the time-kill curve assay and the agar-well diffusion assay support the therapeutic potential of green-synthesized zinc oxide nanoparticles in oral health applications [19]. These nanoparticles exhibit differential antimicrobial activity against a range of oral pathogens, offering versatile solutions for various oral infections.

Furthermore, the study conducted by Hernandez-Delgadillo et al. highlighted the inhibitory effect of zinc oxide nanoparticles on *Streptococcus mutans*, a key contributor to dental caries [20]. This corresponds with the moderate sensitivity of *S. mutans *to zinc oxide nanoparticles observed in the current research. The findings reinforce the potential application of zinc oxide nanoparticles in addressing oral health issues related to bacterial pathogens.

The green synthesis of zinc oxide nanoparticles (ZnONPs) using plant extracts, such as the herbal formulation involving *Ocimum* *tenuiflorum *and *Ocimum gratissimum*, has gained attention in recent years [21]. Patra and Baek's study [22], which utilized the leaf extract of *Ocimum tenuiflorum,* reported excellent antimicrobial activity of green-synthesized zinc oxide nanoparticles against various pathogens. This supports the methodology employed in the present study and underscores the potential of these herbal extracts for eco-friendly nanoparticle synthesis. In another study by Rahman et al., nanoparticles of ZnO, MgO, NiO, AlO, and composite oxides were synthesized utilizing an extract derived from Ocimum basilicum leaves [23]. The antibacterial properties of these nanoparticles were subsequently examined. Notably, zinc oxide nanoparticles demonstrated a pronounced inhibitory effect against *Pseudomonas aeruginosa*. This demonstrates the versatility of plant-based approaches for the eco-friendly production of zinc oxide nanoparticles [24].

Overall, the herbal formulation-mediated synthesis of zinc oxide nanoparticles using *Ocimum tenuiflorum* and *Ocimum gratissimum* represents a promising approach to addressing oral infections. The comprehensive antimicrobial assessment through both time-kill curve and agar well diffusion assays highlights the potential of green-synthesized zinc oxide nanoparticles as effective tools in oral healthcare, with varying sensitivity across oral pathogens, providing a foundation for tailored treatment strategies [25]. Further exploration in this field may unlock the full potential of zinc oxide nanoparticles as valuable assets in oral health management.

Limitation

The study primarily focuses on efficacy, lacking a detailed exploration of the underlying mechanisms. Optimization challenges, including achieving an optimal balance between efficacy and safety, are mentioned but not extensively explored. The narrow focus on a panel of oral pathogens, coupled with the reliance on single assay methodologies, limits the generalizability of findings to a broader spectrum of microbial challenges encountered in clinical scenarios. These limitations underscore the need for further in vivo research to address these gaps and enhance the overall robustness and translational applicability of our findings.

## Conclusions

The study investigated the antimicrobial activity of green-synthesized zinc oxide nanoparticles using an herbal formulation containing Ocimum tenuiflorum and Ocimum gratissimum against a spectrum of oral pathogens, which holds substantial translational value for the field of oral healthcare. 1) The results of both the time-kill curve assay and the agar well diffusion assay provided valuable insights into the antimicrobial efficacy of ZnONPs. 2)The agar-well diffusion assay indicated uniform zones of inhibition (9 mm) for all tested organisms across various ZnONP concentrations; the time-kill curve assay revealed variations in sensitivity among these microorganisms. *C. albicans* demonstrated the highest sensitivity to ZnONPs, followed by *S. mutans* and *S. aureus*, which displayed moderate sensitivity. In contrast, *E. faecalis* and *Lactobacillus* species exhibited lower sensitivity, suggesting the potential for selective targeting of pathogenic bacteria while sparing beneficial ones.

These findings collectively underscore the potential of green-synthesized ZnONPs as versatile antimicrobial agents in oral health applications. The stable and consistent antimicrobial activity observed in the agar-well diffusion assay indicates the reliability of these nanoparticles. The translational value of this research extends beyond the laboratory, providing a foundation for future investigations and potential clinical applications. Overall, this study represents a remarkable step forward in harnessing the potential of green-synthesized zinc oxide nanoparticles as versatile and effective antimicrobial agents with promising translational implications for oral healthcare and biomedical applications.
